# Understanding the Integrated Pathways and Mechanisms of Transporters, Protein Kinases, and Transcription Factors in Plants under Salt Stress

**DOI:** 10.1155/2021/5578727

**Published:** 2021-04-12

**Authors:** Wasifa Hafiz Shah, Aadil Rasool, Seerat Saleem, Naveed Ul Mushtaq, Inayatullah Tahir, Khalid Rehman Hakeem, Reiaz Ul Rehman

**Affiliations:** ^1^Department of Bioresources, Faculty of Biological Sciences, University of Kashmir, Hazratbal, Srinagar, India; ^2^Department of Botany, Faculty of Biological Sciences, University of Kashmir, Hazratbal, Srinagar, India; ^3^Department of Biological Sciences, Faculty of Science, King Abdulaziz University, Jeddah, Saudi Arabia

## Abstract

Abiotic stress is the major threat confronted by modern-day agriculture. Salinity is one of the major abiotic stresses that influence geographical distribution, survival, and productivity of various crops across the globe. Plants perceive salt stress cues and communicate specific signals, which lead to the initiation of defence response against it. Stress signalling involves the transporters, which are critical for water transport and ion homeostasis. Various cytoplasmic components like calcium and kinases are critical for any type of signalling within the cell which elicits molecular responses. Stress signalling instils regulatory proteins and transcription factors (TFs), which induce stress-responsive genes. In this review, we discuss the role of ion transporters, protein kinases, and TFs in plants to overcome the salt stress. Understanding stress responses by components collectively will enhance our ability in understanding the underlying mechanism, which could be utilized for crop improvement strategies for achieving food security.

## 1. Introduction

Plants frequently encounter unfavourable abiotic stresses like extreme temperatures, drought, waterlogging, contamination of soils by heavy metals (HMs), and high salt concentrations. These factors have been recurrently reported to drastically impact agricultural productivity which might be reduced ≥50% for main crops [1]. Among the stresses, salinity is considered most deteriorating as it affects ~20% of irrigated agricultural land and one-third of the agricultural productivity around the globe [2]. The total area under salinization is continuously increasing as it is predicted that by the year 2050, more than half of the land will be salinized [3]. Salinity induces osmotic stress, ionic stress, oxidative stress, imbalance of nutrients, and membrane disorder and reduces cell division [4]. Water deficiency mediated by the increased efflux of water from the root cells leads to osmotic stress. Ionic stress arises due to disproportionate influx of Na^+^ ions via root cell which disturbs the Na^+^/K^+^ and Na^+^/Ca^2+^ equilibrium. This results in increased Na^+^, decreased K^+^ and Ca^2+^ concentrations which causes destabilization of the cell membranes, obstruction of enzymatic activities, and inhibition of normal functioning of the cell [5]. Consequently, there is an overproduction of reactive oxygen species (ROS) like O^2-^ (superoxide radical), H_2_O_2_ (hydrogen peroxide), O_2_ (singlet oxygen), and OH^−^ (hydroxyl ions) in the cytosol, mitochondria, and chloroplast [6]. ROS production in excess is destructive to the cell as it disrupts membranes, mutates DNA, and degrades lipids, proteins, and photosynthetic pigments [7]. It affects photosynthesis by hampering chloroplastic functions and stomatal closure [8]. Plants gauge stress cues and transmit specific stress signal to elicit cellular as well as molecular response as they possess an inbuilt mechanism for adaptations at cellular, tissue, and organ levels. The adaptation includes osmoregulation/osmotic adjustment, ion homeostasis, ion compartmentalization, stomatal regulation, antioxidative defence mechanism, accumulation/exclusion of toxic ions, and changes in morphology, anatomy, and the hormonal profile [9]. Likewise, plants also regulate several genes or their products either vital metabolic proteins or other regulatory genes which confer stress tolerance. These genes are categorized into 2 groups. The first group comprises of genes regulating protein channels, membrane transporters responsible for active/passive transport, detoxification of enzymes, enzymes responsible for fatty acid metabolism, protease inhibitors, and enzymes responsible for overproduction and accumulation of compatible solutes, LEA (late embryogenesis abundant) protein, osmotin, and chaperons. The second group of genes is responsible for regulatory proteins (transcription factors (TFs) protein kinases and protein phosphatases) which respond to the signals downstream and modulate the expression of related genes [10]. In this review, we aim to discuss salt stress sensitivity, the role of ionic transporters, and the related regulatory gene products that allow the plants to alleviate salt stress at the cellular and/or molecular level. It focuses on protein kinases and TFs associated with salt stress tolerance and illustrates their potential for crop improvement.

## 2. Consequences of Salinity

Plants are grouped into halophytes and glycophytes, based on their capability to thrive in saline environments. The former group has resistance mechanisms to withstand higher salt concentrations, while the latter group lack such mechanisms. The difference in behaviour between them is attributed to their variation in the photosynthetic electron transport chain, assimilation of CO_2_, photosynthetic pigment content, ROS generation, and sequestration [11]. Salinity primarily generates osmotic, ionic, and oxidative stress, which alters the morphological, physiological, and molecular aspects of plants, thereby affecting their overall metabolism and growth [12]. The osmotic stress causes water deficit by increased water efflux due to increased Na^+^ influx resulting in damage to photosynthetic apparatus by disrupting the thylakoid membrane and Calvin-Benson cycle enzymes [13], resulting in reduction of specific metabolites. The accumulation of Na^+^ and Cl^−^ ions causes reduction of specific metabolites which gives rise to nutrient deficiency [14]. This is followed by an overproduction of ROS (oxidative burst) [15, 16], which prompts damage to nucleic acids, proteins, and lipids. In DNA, they cause mutations, deletions, inhibition of replication, transcription, and signal transduction. In proteins, they cause susceptibility to proteolysis, variation in amino acid profile, chain fragmentation, and accumulation of cross-linked reaction products. In lipids, they initiate spontaneous oxidative chain reactions on unsaturated fatty acids. Thus, ROS destabilizes plasma membrane by inducing lipid peroxidation and protein disintegration, resulting in its impaired integrity [17].

## 3. Salinity Perception and Response

**Figure 1:**
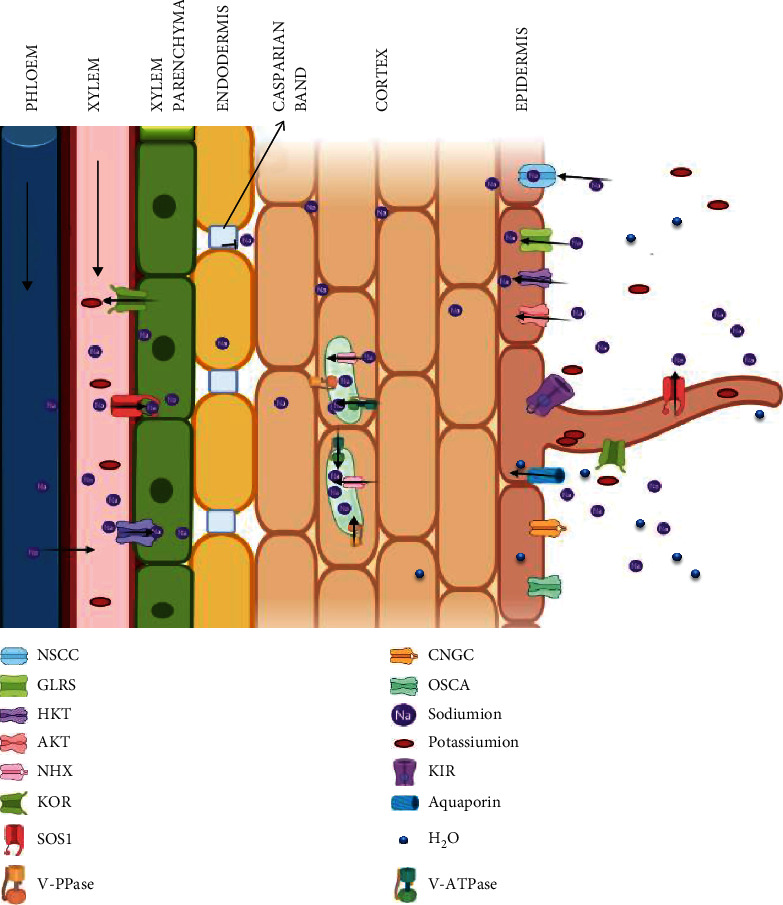
Schematic representation of *Na^+^* and *K^+^* transporters mediating Na^+^ and K^+^ homeostasis in plant roots under salt stress. Na^+^ ions enter the cells via nonselective cation channels (NSCCs) and possibly via other cation transporters (symplast flow) and intercellular spaces (apoplast flow). The SOS1 extrudes Na^+^ at the root-soil interface and the xylem parenchyma cells. Likewise, HKT also retrieves Na^+^ from the xylem. SOS1, localized in the xylem parenchyma cells, mediate Na^+^ efflux from xylem vessels under high salinity. Excessive Na^+^ in root is sequestered in the large central vacuole by tonoplast-localized NHX exchangers, V-ATPase, and V-PPase which also generate electrochemical potential gradient for secondary active transport.

Plants perceive innumerable environmental signals which initiate response mechanisms. Plant cells communicate constantly to coordinate activities in response to hypersaline environment by employing various signalling cascades. Plasma membrane acts as a physical barrier at the root-soil boundary. It is impermeable to hydrophilic molecules like ions, water, and macromolecules but permeable to small lipophilic molecules like steroid hormones. However, hydrophilic macromolecules are transported through different channels or carriers. Upon exposure to the saline environment, the initial reaction may relay within a few seconds or may take hours. The nonselective cation channels (NSCCs), glutamate receptors (GLRs), high-affinity K^+^ transporters (HKTs) and K^+^ channels like *Arabidopsis* K^+^ transporter (AKT1), and high-affinity K^+^ uptake transporter (HAK) of root epidermal cells are responsible for Na^+^ influx, which further inhibits inward rectifying K^+^ channels and activates K^+^ outward-rectifying channels (KOR) [18, 19]. Furthermore, under saline conditions, aquaporins are also believed to import Na^+^ from soil which results in osmotic stress [20]. This stress is perceived by PM's mechanosensitive receptor proteins which communicate the signal by accumulating cGMP leading to calcium (Ca^2+^) accumulation. Furthermore, secondary messengers like diacylglycerol (DAG), inositol phosphates (IPs), and ROS are also produced immediately after perception. For relaying the response downstream and modulating stress-responsive genes, different salt-responsive pathways, viz., salt overly sensitive (SOS), protein kinase, Ca^2+^, ABA (abscisic acid), and other phytohormones, are involved. The responsive genes are grouped into two categories as early and late induced genes. Early induced genes comprises TFs expressed rapidly as soon as the stress signal is relayed while the late genes like stress-responsive genes are activated slowly in hours after stress perception. For early genes, the signalling components are already primed but the late genes which have sustained expression encode and modulate the required proteins, e.g., RD (responsive to dehydration). These gene products augment the primary signal and induce a second round of signalling, which may follow the previous pathway or opt for a new signalling pathway. An overview of the initial signalling responses is presented in Figure 1.

Plants respond to salt stress by different mechanisms, and ABA-signalling is considered its principal regulating pathway [21]. Various other mechanisms like membrane system adjustment, cell wall modifications, variations in cell division, cell cycle, and alteration in metabolism operate in either isolation or synchronization to overcome the adverse effects of salinity. Ion homeostasis comes into play to limit the excess accumulation of Na^+^, maintaining the water flux, and K^+^ concentration [22]. Similarly, to sustain low osmotic potential, plants synthesize and accumulate organic compounds known as osmolytes or compatible solutes such as polyols, nonreducing sugars, and nitrogen-containing compounds. Osmolytes protect the important proteins by excluding the hydrophilic molecules from their hydration sphere so that their interaction with water is reduced or inhibited. Therefore, their native structures are protected and thermodynamically favoured. Another important key event in plants is epigenetic regulation of stress-inducible genes which helps in adaptation, wherein a particular gene is either constrained or overexpressed by modification of DNA-associated proteins or the DNA itself.

## 4. Ion Homeostasis

**Figure 2:**
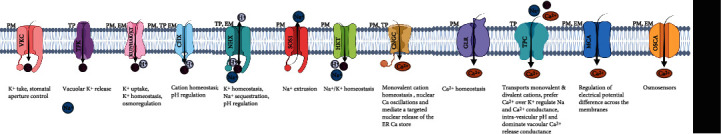
Picture depicting different transporters and channels with functions responsible for ion homeostasis under salt stress. These transporters and channels are found on plasma membrane (PM), tonoplast (TP), and endomembranes (EM). K^+^ homeostasis-related transporters/channels include voltage-dependent K^+^ channel, two-pore K^+^ channel (TPK), K^+^ uptake permease/high-affinity K^+^/K^+^ transporter (KUP/HAK/KT), and cation/H^+^ exchanger (CHX). Na^+^ homeostasis-related transporters/channels include Na^+^/H^+^ exchanger (NHX), salt overly sensitive 1 (SOS1), and high-affinity K^+^ transporter (HKT). Ca^2+^-related transporters/channels include cyclic nucleotide-gated channels (CNGCs), glutamate receptor-like channels (GLRs), two-pore channels (TPCs), mechanosensitive channels (MCAs), and reduced hyperosmolality-induced Ca^2+^ increase channels (OSCAs).

Plants regulate Na^+^ concentration by exclusion, redistribution, elimination, succulence, and accumulation in the cytoplasm until its osmotic potential is lower than the soil. Plasma membrane along with its channel proteins, antiporters and symporters, plays a significant role in transport and balancing of cytosolic ion concentration. The important step in the initiation of ion homeostasis is holding back the excess accumulation of Na^+^/K^+^ and maintaining the water flux [23]. Both glycophytes and halophytes cannot withstand ion toxicity in their cytosol and thus transport excessive salts to the vacuoles or sequester them into the older leaves and tissues [22]. Under the saline condition, the Na^+^ enters the plant passively through root endodermis or by various channels NSCCS, GLRs, and HKTs [24]. Major transporters involved in attaining Na^+^ homeostasis are the SOS1 antiporter in the root for Na^+^ efflux to soil, NHX antiporters for Na^+^ sequestration into the vacuoles, and HKT transporters to retrieve Na^+^ from the transpiration stream (Figure 1). Plants possess different transporters which work in tandem to protect the plant from the adverse effect of Na^+^ accumulation (Figure 2).

### 4.1. Salt Overly Sensitive (SOS) Pathway

Roots are the primary site of salt stress perception, and the plasma membrane consists SOS1 as the main transporter of Na^+^ which is involved in its extrusion [25]. SOS pathway comprises *SOS1*, *SOS2*, and *SOS3* genes which regulates Na^+^ homeostasis. The *SOS3* encodes a small protein with Ca^2+^ binding and myristoylation sequence (MGXXXST/K) for its activity by aiding the protein–protein and protein–lipid interactions. In plants, *SCaBP8/CBL10* (a paralog of SOS3) is equivalently expressed in the shoots and is a Ca^2+^-binding and calcineurin B-like (CBL) protein. SOS3 protein kinase senses the modulated level of cytosolic calcium elicited by salt stress. It forms a complex with serine/threonine-protein kinase encoded by *SOS2*. The SOS2 comprises C-terminal regulatory domain with FISL/NAF motif of 21amino acid and the N-terminal catalytic domain, which shares sequence homology with SNF (sucrose nonfermenting) kinases [26]. Under normal circumstances, FISL motif interacts with the catalytic domain for autoinhibition. However, during stress condition, SOS2 is activated by calcium-dependent SOS3 through its regulatory domain (FISL motif) by relieving it from an autoinhibition mode. [27]. Deletion of FISL motif from SOS2 activates it constitutively to make its expression independent of SOS3 [28]. The SOS1 is activated by the SOS3–SOS2 complex, by myristoylated N terminus motif of SOS3 [29]. SOS1 is a Na^+^/H^+^ exchanger which transports the Na^+^ ions from root epidermal cells into xylem parenchyma cells for transport up to leaves [21] while meristematic root tip cells lack vacuoles and possess SOS1 in their epidermis for extruding Na^+^ into the soil [22]. Various studies under salt stress employing wild types and mutants deficient in *SOS1*, *SOS2*, and *SOS3* genes have demonstrated that all these are vital to improve salt stress [30].

SOS2 also sequesters excess Na^+^ ions into the vacuoles through vacuolar ATPases by binding to their regulatory units and influence the Na^+^/H^+^ exchange [22]. Tonoplast comprises two types of antiporters, viz., vacuolar-type H^+^-ATPase (V-ATPase) and vacuolar pyrophosphatase (V-PPase) [31]. Under stress conditions, V-ATPase is considered more responsible for the survival of plant by sequestering Na^+^ into vacuoles [32]. In *Vigna unguiculata*, the V-ATPase activity has been reported to increase under salinity, while it remains inactive under normal conditions [33]. In *Arabidopsis*, salinity tolerance has been reported to be independent of V-ATPase activity, as the loss of V-ATPase function did not change the salinity tolerance. However, a direct relationship between H^+^-ATPase of transgolgi network and salt stress response has been reported in plants [34]. It has been also reported that the mutation of transgolgi network-specific marker genes, viz., *V-ATPase subunit VHA-a* (*VHA-A1*), *SYNTAXIN OF PLANTS 61* (*SYP61*), *RAB GTPases A Group 2A* (*RABA2A*), or *SYNTAXIN OF PLANTS 43* (*SYP43*), results in salt sensitivity in different plants [35]. *Arabidopsis* having *tno1* (*tgn-localization syp41-interaction protein*) mutants has irregular localization of SYP61 and is sensitive to salt stress [36].

Interestingly, the overexpression of V-PPase has been reported to improve the salt tolerance in plants by facilitating the vacuolar Na^+^ sequestration [37]. The effect of H^+^-PPase in crop plants (by overexpressing AtAVP1) in *Hordeum vulgare* has been reported, not only to increase salinity tolerance under greenhouse gases but also to improve the grain yield along with improved shoot biomass [38]

## 5. Sodium-Hydrogen Exchanger Proteins (NHX)

Sodium-hydrogen exchanger proteins (NHXs) are the transporters involved in cell expansion, ion homeostasis, and salt tolerance which catalyze the electroneutral exchange of K^+^ or Na^+^ for H^+^ [39]. NHXs sequester Na^+^ by ATP-dependent transport under saline conditions [40]. There are eight NHXs (AtNHX1-8) in *Arabidopsis*, which are categorized into three groups: Group I (AtNHX1–4) present on vacuolar membranes, Group II (AtNHX5-6) localized Golgi apparatus and endosomes, and Group III (NHX7/SOS1 and NHX8) on plasma membrane [41]. Overexpression of AtNHX1 and AtNHX2 in *Arabidopsis* and AtNHX1 in tomato, rapeseed, and soybean is reported to elicit a salt response and confer tolerance [5, 42–44]. Similarly, overexpression of heterologous NHX from *Pennisetum glaucum* conferred tolerance in tomato [45], and overexpression of *NHX* from halophyte *Suaeda salsa* and *Arabidopsis* respectively increased salt tolerance in transgenic rice and cotton [46]. Furthermore, *OsNHX1*, a homolog of AtNHX1, expresses in root hairs and guard cells in aerial parts under salinity stress to confer tolerance by storing Na^+^ in their vacuoles [47].

## 6. High-Affinity K^+^ Transporters HKT

They are important Na^+^ carriers which are grouped into class I HKT transporters specific for Na^+^ in both monocots and dicots and class II HKT symporters having an affinity for Na^+^ and K^+^ in monocots [48]. Moreover, HKTs show 26-fold higher affinity towards the Na^+^ than K^+^ in saline conditions [49]. It is an important long-distance Na^+^ transporter located in xylem parenchyma in the vascular bundles all over the plant. HKT1 retrieves the Na^+^ from the xylem into xylem parenchyma inhibiting its delivery into the leaf [50]. To adapt under salinity, some of the Na^+^ reaches leaf tissue from xylem where it is translocated into the phloem, from where it travels back to the roots to reduce its levels in shoots as reported in corn, pepper, and barley [50]. *In vivo* electrophysiological analyses of the root, stellar cells from *Arabidopsis* mutant and wild type showed that HKT1 mediates passive Na^+^ transport [51]. Similarly, OsHKT1;5 an ortholog of HKT1;1 also has a role in sequestering Na^+^ from xylem to xylem parenchyma to protect the aerial parts of plant, and TaHKT1;4 transformation resulted in improved tolerance and yield [52]. AtHKT1;1 is also known to elicit the indirect xylem loading of K^+^ via outward-rectifying K^+^ channels to maintain high K^+^/Na^+^ ratio in leaves to neutralize Na^+^ stress [53]. Mutation of AtHKT1;1 and OsHKT1;4 in *Arabidopsis* and rice resulted in Na^+^ hypersensitivity due to Na^+^ accumulation in leaves [54, 55].

## 7. K^+^ Homeostasis

The most abundant cation K^+^ plays various roles such as osmotic homeostasis, protein translation, sugar transport, and photosynthesis. Generally, the cytoplasmic concentration of Na^+^ is maintained at less than 1 mM, while the K^+^ accumulates up to 100 mM. The ability of plant tissues to retain potassium under stress have emerged as important for salinity tolerance, but recent evidence suggests that stress-induced K^+^ efflux may be equally important in mediating growth and development under hostile conditions [56]. Cellular K^+^ level is maintained by various channels and transporters located at different interfaces including transporters at the root-soil interface, xylem loading, and vacuolar membranes. Various channels responsible at root-soil interface are *Arabidopsis* shaker type (AKT), high-affinity potassium transporter (KUP/HAK), cyclic nucleotide-gated channel (CNGC), K^+^ release channel, and guard cell outward-rectifying K^+^ channel (GORK). Xylem possesses selective K^+^ channel, viz., Stelar outward-rectifying channel (SKOR), nonselective cation channels (NSCC) while as phloem possesses AKT. K^+^ accumulation in vacuoles is driven by H^+^-coupled antiporters such as NHX, while the release is mediated by K^+^ channel called the tonoplast two-pore K^+^-type channel (TPK1). Uptake and transport mechanism of K^+^ predominantly depend on the available concentration of K^+^ in soil. But, when the extracellular concentration of Na^+^ is high compared to the concentration of K^+^, Na^+^ is preferred by the transporters because of their similar charge resulting in reduced K^+^ uptake [24]. However, K^+^ deficiency is secured by root hair and epidermal cells where the signal is transduced to the cytosol [4]. K^+^ transporters facilitate high-affinity K^+^ uptake than K^+^ channels for maintaining K^+^ homeostasis. Conversely, when the extracellular concentration of K^+^ is more, K^+^ channels facilitate low-affinity K^+^ uptake for maintaining homeostasis [57]. In root tissues, K^+^ either accumulates locally in vacuoles or is transported to aerial parts through the xylem. The excessive K^+^ surpassing the nutritional requirements is accumulated in the vacuoles generates turgor pressure and aids in cell expansion. Under the initial stages of water deficit in plant, K^+^ subsidizes the osmotic adjustment till the compatible solutes are made available. Accumulation of K^+^ has been proved to play a considerable part in salt tolerance by maintaining the Na^+^/K^+^ ratio, turgor pressure, and accumulation of osmolytes. Exogenous application of K^+^ has proved to confer increased salt stress tolerance in Lucerne, barley, wheat, and canola [42].

## 8. Ubiquitous Ca^2+^ Transporters

Calcium (Ca^2+^) is the ubiquitous secondary messenger which coordinates different plant responses against various environmental cues. Ca^2+^ involves 5 different types of transporters: cyclic nucleotide-gated channels (CNGCs) in PM and tonoplast, glutamate receptor-like channels (GLRs) in PM, two-pore channels (TPCs) in tonoplast, mechanosensitive channels (MCAs), and reduced hyperosmolality-induced Ca^2+^ increase channels (OSCAs) in PM and endomembranes. In response to salinity, the cytosolic concentration of Ca^2+^ increases, which is transported from distinct sites to the cytoplasm [58]. To decode an increased level of Ca^2+^, cells possess specific tools and mechanisms that include Ca^2+^ sensors and target proteins. The sensor proteins possess a Ca^2+^-binding site in their helix-loop-helix region and are classified into two categories as sensor responders and sensor relays. Sensor responders such as Ca^2+^-dependent protein kinases (CDPKs) exhibit both Ca^2+^ binding and kinase activity, while sensor relays like calmodulin (CaM) and calmodulin-like proteins (CML) do not contain kinase activity. However, after binding with Ca^2+^, they interact with other protein kinases to regulate their activities [59]. This increased concentration of Ca^2+^ activates CaM, CML, CDPK, and CBLs which play a pivotal role in signal transduction. The CaM proteins activated by Ca^2+^ initiates the signalling cascade via the calcineurin pathway involving the CDPK, which further modulate the calcium transporters and regulate the ion transport [60, 61]. In rice, an increased expression of *OsCam1–1* under saline stress showed better growth than its corresponding wild type [62, 63]. Overexpression of *GmCaM4* (*Glycine max* calmodulin) in *Arabidopsis* resulted in expression of *AtMYB2*-regulated genes including genes for proline biosynthesis resulting in proline accumulation which confers salt tolerance [64]. Calcineurin B-like-interacting protein kinase (CIPK) forms a complex with CBL, which further interacts with other proteins like SOS1 and AKT1 to regulate their function to help attain ion homeostasis [65]. Studying the Ca^2+^ increase in relation to salt stress led to the identification of *monocation-induced Ca^2+^ increases1* (*moca1*) mutant, lacking the Ca^2+^ increase induced by Na^+^; however, it remained unaffected by other multivalent cations, ROS, or osmotic stress [66].

## 9. Role of Protein Kinases in Response to Salt Stress

Diverse protein kinases in plants play a significant role in integrating different stress-signalling pathways which are responsible for combating the adverse effects of salinity. Mitogen-activated protein kinase (MAPK) cascade is one of the prime pathways in sensing the osmotic stress caused by salinity and transducing it downstream. MAPK pathway comprises MAPKKK, MAPKK, and MAPK which are present in the nucleus and cytoplasm and are linked to downstream targets and the receptors. The MAPK pathway receptor activation takes place by its phosphorylation by receptor itself, by interconnecting MAPKKKKs, by linking factors and/or by physical interaction with certain compounds. The MAPKKs are dual-specificity kinases which are phosphorylated at two serine/threonine residues of a conserved S/T–X_3–5_–S/T motif. These MAPKKs further phosphorylate MAPKs, a serine/threonine kinases at threonine and tyrosine residues in the T–X–Y motif. These MAPKs are responsible for phosphorylation of a variety of substrates including regulatory proteins like TFs, kinases, and cytoskeleton-associated proteins [67]. In response to osmotic stress, the transcript level for these MAPKs increases which ultimately leads to accumulation of compatible solutes for reestablishment of osmotic balance in cell and induces the major stress genes like LEA/dehydrin for protection from stress damage [25]. On the onset of salt stress, different MAPKs mainly MPK4 and MPK6 are stimulated within diverse periods, and MPK3 is activated by osmotic stress [68, 69]. Similarly, various other MAPKs are activated in response to osmotic stress known as SIMK (salt stress-inducible MAPK), and a SIMK-like MAP kinase named SIPK (salicylic acid-induced protein kinase) in alfalfa and tobacco [70–72]. In osmotic stress conditions, MKK4 is reported to accumulate ROS, regulate the activity of MPK3, and target *NCED3* (*NINE-CIS-EPOXYCAROTENOID DIOXYGENASE 3*) of ABA biosynthetic process [36]. Various reports suggest MPK6, MKK1, and MKKK20 results in accumulation of ROS for signal transduction purpose [73]. MPK6 is also proved to directly mediate the phosphorylation of SOS1 by salt in plant sense response [74]. MAPK is linked to ROS signalling via SERF1 (*salt-responsive* ERF1) transcription factor [75].

Another group of kinases are ABA-associated sucrose nonfermenting 1/SNF1-related protein kinase 2 (SnRK2) which mediate in different processes of plant cellular signalling. These SnRK2/OST1 kinases are activated by autophosphorylation and in turn phosphorylate its direct substrates like downstream effector proteins [76]. The strongly activated ABA-SnRKs include SnRK2.2, SnRK2.3, and SnRK2.6/OST1 while SnRK2.7 and SnRK2.8 are weakly activated [77]. ABA-activated SnRK2s induce SLAC1 (slow anion channel-associated1) in plasma membrane under salt stress, which facilitate the water retention and reduce water loss due to transpiration by mediating stomatal closure [78]. SnRK2-mediated phosphorylation of RbohF (respiratory burst oxidase homolog protein F) and NADPH oxidase of plasma membrane results in generation of O^2−^, which is subsequently converted into H_2_O_2_ in apoplastic space. This H_2_O_2_ acts as a signalling molecule and facilitates different stomatal closure with other ABA responses [79]. It is reported that SnRK2.8 directly interacts with transcription factor NTL6 of NAC (NAM/ATAF1/2/CUC2) under the influence of ABA, which controls the cellular functions of abiotic stress [73]. Another study on *Arabidopsis snrk2.2/2.3/2.6* triple-mutant with dwindled ABA sensitivity identified the SnRK2 phosphorylation targets including signal transduction proteins [80]. These progressive research reports the intricate cross talk of SnRK2 kinases with other stress-responsive processes in different plant signalling pathways.

Calcium-dependent protein kinases (CDPKs/CPKs) respond to elevated concentrations of calcium due to different environmental cues. The CDPKs regulate the stomatal movement for maintaining ion homeostasis. So far, 34 CDPKs have been identified in *Arabidopsis*, out of which 27 contain N-myristoylation motifs highlighting their role in membrane-associated processes. Different CDPKs are reported to play a pivot role in ion transport regulation. They have been reported to link the membrane transport to ABA-signalling under water deficit conditions in guard cells. In *Arabidopsis*, AtCPK3 and AtCPK27 are reported to confer salt tolerance [81, 82]. Besides regulating ion transport, CDPKs have a role in ABA and salt stress responses via interacting with diverse proteins and their phosphorylation. OsCPK14 and OsCPK21 in rice are reported to interact with and phosphorylate OsDi19–4 transcription factor and 14-3-3 protein (OsGF14e) respectively [83, 84]. Certain CDPKs also modulate salt stress through osmotic adjustment like OsCPK9 transcripts which were induced by salt treatments [85] In rice, OsCPK10 protein modulate the catalase activity to detox the H_2_O_2_, which further protects the cell membrane integrity [86]. OsCPK12 also regulates ROS homeostasis by inducing the ROS scavenger genes OsAPX2/OsAPX8 and repressing NADPH oxidase gene OsRBOHI and confers salt tolerance [87]. Similar group of calcium-dependent kinases in plants include calcineurin B-like- (CBL-) interacting protein kinases (CIPKs). CBLs are a family of small proteins (~200 amino acid), which perform the regulation of CIPKs. CIPK network plays a vast and pivotal role in ion transport. CIPK functions are well characterized by CIPK24 (SOS2) along with CBL4 (SOS3), which together activates the Na^+^/H^+^ antiporter (SOS1) to improve salinity tolerance [88]. Similarly, CBL1, CBL9, CIPK1, CIPK2, CIPK25, CIPK26, and C(89)IPK31 are reported to mediate the response against salt stress via ABA-signalling [89].

## 10. Transcription Factors and Stress Response

Transcriptomic analysis of different plants suggests their genetic and transcriptional dependent susceptibility and tolerance towards different stresses [90, 91]. Stress-responsive transcription factors (TF) have attained extensive consideration as they not only regulate gene expression but also play a pivot role in regulating multiple abiotic stress responses like salt sensory pathways [92, 93]. TFs regulate downstream stress-responsive gene by binding to *cis*-regulatory elements in their promoter region [94]. They serve as molecular switches to the associated genes by binding to their *cis*-element under different cellular conditions. The chief trait of TF is to interact with different proteins in transcriptional complexes and regulate the expression of a vast number of genes. Nearly, 10% of genes in plants potentially code for TF which are categorized based on their distinct structure of DNA-binding domain [95]. The transcription factors associated with salinity are summarized in Table 1.

### 10.1. NAC

NAC TFs are the largest plant-specific derived from three proteins, viz., NAM, ATAF, and CUC, which possess a conserved DNA-binding domain, and these comprise of diverse C-terminal transcriptional regulatory region as well as N-terminal at C-terminal DNA-binding domain [135]. Overexpression of NAC factors has been reported to assist in achieving improved salt tolerance in many plants like *Arabidopsis*, rice, chickpea, tomato, and chrysanthemum by regulating stress-responsive genes and enhanced physiological activities [100, 136–138]. It is reported that transgenic plants overexpressing SNAC3 showed lower levels of H_2_O_2_, malondialdehyde (MDA) and relative electrolyte leakage compared to the wild type under saline stress [139]. NAC-related genes in several plants such as *Sorghum* (*SbNAC6*, *SbNAC17*, *SbNAC26*, *SbNAC46*, *SbNAC56*, *SbNAC58*, and *SbNAC73*) and wheat (*TaNAC47*) are induced by salt [140, 141]. Another wheat gene, *TaNAC47*, is known to induce downstream genes like *AtRD29A*, *AtRD29B*, and *AtP5CS1* in *Arabidopsis* which alleviate the stress by increasing the osmolytes content. Similarly, overexpression of *TaNAC29*, *EcNAC67*, and *NAC57* from poplar enhanced salt tolerance in transgenic *Arabidopsis* [138, 142, 143].

### 10.2. MYC/MYB

MYC (myelocytomatosis oncogene)/MYB (myeloblastosis oncogene) families are a universal class of protein with highly conserved DNA-binding domains known as MYB domains, which comprises multiple imperfect repeats, and each unit repeat contains approximately 52 amino acids embrace helix–turn–helix (HTH) structure. This HTH intercalates in the major groove of DNA [144]. MYB TFs have potential roles in many physiological processes like in secondary metabolism, cell morphogenesis, meristem formation and floral and seed development, cell cycle control, hormone signalling, defence, and stress responses [105, 145, 146]. *AtMYB2*, *AtMYC2*, *AtMYB73*, *AtMYB77*, *AtMYB41*, *AtMYB44*, *AtMYB102*, and *OsMYB3R-2* are transcriptionally regulated in salt stress, conferred salt tolerance in transgenic plants [104, 147–152].

## 11. AP2/ERF

APETALA2/ethylene response element-binding factors (AP2/ERF) TFs are characterized by specific DNA-binding domain that binds to the GCC box of the DNA [153, 154]. This conserved domain is responsible for multiple functions in plant development like cell proliferation, reproduction, hormone, and stress responses [155]. The AP2/ERF TF family is categorized in 4 subfamilies, viz., DREB (dehydration-responsive element-binding protein), ERF (ethylene response element-binding factors), AP2 (Apetala 2), and RAV (related to ABI3/VP1) [153, 155]. Among these four, DREB and ERF have been comprehensively studied in response to salt stress, and some members of the RAV subfamily have also been reported to modulate salt stress [156]. The distinct DREB subfamily has a substantial part to play in stress regulation [157]. The DREB1/CBF binds to the *cis*-acting elements of stress-responsive genes with conserved sequence (5′-TACCGACAT-3′), which constitute their drought-responsive element (DRE) in the promoter region [158]. DREBs are categorized into two subgroups DREB1 and DREB2 and are induced by dehydration and salt stress [159]. Constitutive expression of DREB1/CBF3 conferred salt tolerance to transgenic plants, like the overexpression of *Suaeda salsa SsCBF4*, confers salt tolerance in transgenic tobacco [160]. Apple *MbDREB1* and wild barley *HsDREB1A* overexpressed in *Arabidopsis* and bahiagrass imparted salt tolerance [161, 162]. DREB2-type proteins are believed to function through a conserved regulatory mechanism in several crops like wheat, maize, rice, and barley [163]. Many DREB2A are induced by high salinity and dehydration like rice *OsDREB2A*, maize *ZmDREB2A*, and *Arabidopsis AtDREB2A* [114, 118, 164]. Transgenic *Arabidopsis* with overexpressing DREB2A-CA exhibits enhanced salt tolerance by modulating the expression of salt-responsive genes [165]. Likewise, overexpression of *PgDREB2A* in transgenic tobacco plants confers tolerance against ion toxicity and osmotic stresses [166].

### 11.1. AREB/ABF TFs

The ABA-responsive element-binding protein/ABA-binding factor (AREB or ABFs) belong to the bZIP (basic leucine zipper) TF group. AREB/ABFs modulate the expression of ABA-responsive genes by binding to their ABA-binding responsive elements (ABREs). These ABREs possess conserved G-box-like *cis*-acting element (PyACGTGG/TC) in their promoter region [167]. AREB/ABF TFs bind either to several ABREs simultaneously or to ABRE along with the coupling element (CEs) like CE1, CE3, DRE/CRT, and motif III [168]. The signalling pathways of these require SnRK2s to regulate the ABA-responsive genes under stress conditions. Under normal conditions and absence of ABA, SnRK2 is dephosphorylated by phosphatases 2C (PP2Cs), and hence, their activity is inhibited. In stress conditions, ABA inhibits the PP2Cs via ABA receptor (PYR/PYL/RCAR) proteins by binding to their regulatory components, viz., Pyrabactin resistance1/PYR1-like [169]. Thus, the SnRKs are activated, which phosphorylates these AREB/ABF TFs. These TFs comprised 4 different conserved domains for phosphorylation by different ABA-activated SnRK2 commonly SRK2D/SnRK2.2, SRK2E/SnRK2.6, and SRK2I/SnRK2.3. These phosphorylated TFs bind to the ABRE *cis*-element and regulate the expression of stress-responsive genes [91, 170].

## 12. Conclusion

**Figure 3:**
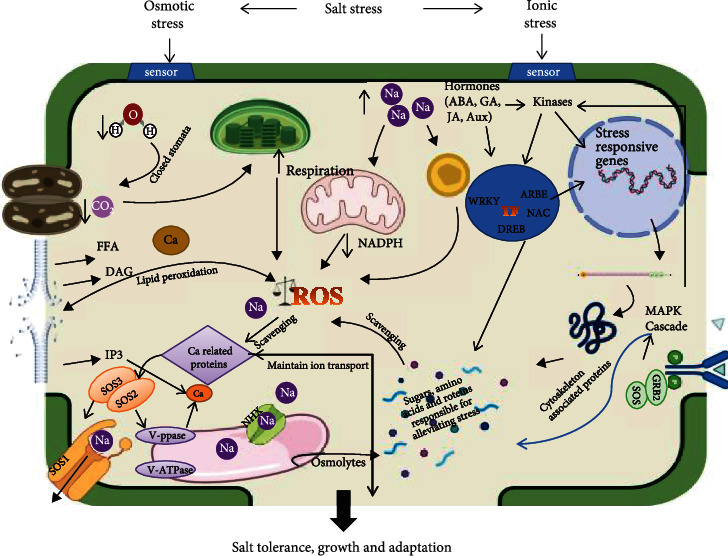
Mechanism of salinity tolerance in plants. The excess influx of sodium causes ion toxicity and water deficit, which results in the closure of stomata and decreased availability of CO_2_ for photosynthetic ETC. This water deficit eventually causes ion imbalance and overproduction of ROS in chloroplast, mitochondria, peroxisomes, and apoplastic space. In response, the plants increase enzymatic/non enzymatic antioxidants and osmolytes. Transporters like NHX sequester Na^+^ inside the vacuole and SOS1 extrudes Na^+^ outside the cell. In this response, there is an increased expression of salt-responsive genes, transcription factors (TFs), and kinases inside the cell, which help the plants to alleviate the stresses encountered by them.

Different signalling components together play an important role in regulating abiotic stress response and play a critical role in conferring stress endurance and tolerance to plants. Usually, the abiotic stress mechanism and the signalling pathways are studied in model plants which provide us with insight into its working (Figure 3). High-throughput sequencing and functional genomics tools have helped in understanding the cross talk between the different components involved in stress-related signalling. There is still insufficient information on abiotic stress-signalling components and their interconnection in alleviating stress. Significant work has been done in interpreting the role of signalling components and their cross talk to achieve tolerance against salinity. Various promising pathways have been elucidated, they need to be envisaged as complex networks, and their cross talk needs to be enlightened. Thus, comprehensive research on the functional architecture of complex networks, including their interactions and cross talk towards abiotic stress, is required for practical exploitation of them in alleviating the abiotic stress. Expression of many of these stress-responsive genes is regulated by TFs in either an ABA-dependent or ABA-independent manner and helps plants to sustain single or multiplicative effects of different abiotic stresses. Different studies on different species of plant have shed light on the intricate and important role of TF in alleviating the abiotic stress. A significant number of TF genes have been identified and validated, but various stress-responsive TF genes, which are proposed to have a considerable role in stress tolerance and connects different signalling components, deserve attention. The cumulative expression of some TF genes may improve the stress tolerance at the cost of growth, flowering, and yield which needs to be addressed. In the future, the focus should be on the novel candidate genes which confer the tolerance in halophytes. Last but not least, focus must be shifted from commercial crops to the nutrient-rich pseudocereals and millets which are promising future crops with high nutritive value.

## Figures and Tables

**Table 1:** List of transcription factors associated with salinity.

Family	DNA-binding domains	*Cis*-acting element	Plant species	Genes involved in salt response	Reference
NAC	NAC domain	NAC recognition sequence (TCNACACGCATGT)	*Arabidopsis*	*AtNAC2* *AtNAC019* *AtNAC055* *AtNAC072*	[46, 96]
			*Oryza sativa*	*OsNAC6* *SNAC1* *SNAC2*	[97–99]
			*Cicer arietinum*	*CarNAC5*	[100]
			*Triticum aestivum*	*TaNAC4*	[101]
			*Gossypium hirsutum*	*GhNAC4* *GhNAC6*	[102]
			*Setaria italica*	*SiNAC*	[103]

MYB	MYB domain	MYBR (TAACNA/G)	*Arabidopsis*	*AtMYB2 AtMYB4* *AtMYB6* *AtMYB7* *AtMYB44* *AtMYB73* *MYB15*	[104–107]
			*Glycine max*	*GmMYB76 GmMYB92*	[108]

WRKY	WRKYGQK domain	W-box (TTGACT/C)	*Oryza sativa*	*OsWRKY45*	[109]
			*Nicotiana benthamiana*	*NbWRKY*	[110]
			*Glycine max*	*GmWRKY21 GmWRKY54 GmWRKY13 GmMYB177*	[108, 111]

ERF/DREB	AP2/ERF domain	DRE sequence, GCC box (AGCCGCC), and (TACCGACAT)	*Arabidopsis*	*DREB2A* *DREB2C*	[112, 113]
			*Oryza sativa*	*OsDREB1A OsDREB1C OsDREB1F* *OsDREB2A*	[114, 115]
			*Hordeum vulgare*	*HvDRF1* *HvDREB1*	[116, 117]
			*Zea mays*	*ZmDREB2A*	[118]
			*Pennisetum glaucum*	*PgDREB2A*	[119]
			*Setaria italica*	*SiDREB2*	[120]
			*Capsicum annum*	*CaDREBLP1*	[121]
			*Artiplex hortensis*	*AhDREB1*	[122]
			*Glycine max*	*GmDREBbGmDREBc* *GmDREB2*	[123, 124]
			*Dendronthema* x *moriforlium*	*DmDREBa*	[125]
			*Cicer arietinum*	*CAP2*	[126]
			*Salicornia brachiata*	*SbDREB2A*	[127]

bZIP	bZIP domain	GLM (GTGAGTCAT), ABRE (CCACGTGG), GCN4-like-motif (GTGAGTCAT), C-box (GACGTC), A-box (TACGTA), G-box (CACGTG), PB-like(TGAAAA), GLM (GTGAGTCAT	*Arabidopsis*	*ABF2* *ABF3* *ABF4*	[128]
			*Glycine max*	*GmbZIP44* *GmbZIP62* *GmbZIP78* *GmbZIP132*	[129]
			*Triticum aestivum*	*Wlip19*	[130]
			*Oryza sativa*	*OsABI5* *OsbZIP23*	[131, 132]
			*Zea mays*	*ZmbZIP17*	[133]
			*Solanum lycopersicum*	*SlAREB*	[134]

## Abbreviations

ABA: Abscisic acid
ABFs: ABA-binding factor
AKT1: Arabidopsis K^+^ transporter
AP2/ERF: APETALA2/ethylene response elementbinding factors
AREB: ABA-responsive element-binding protein
Ca^2+^: Calcium
CBL: Calcineurin B-like
CDPKs/CPKs: Calcium-dependent protein kinases
cGMP: Cyclic guanine monophosphate
CIPKs: CBL-interacting protein kinases
Cl^−^: Chlorine
CNGC: Cyclic nucleotide-gated channel
DAG: Diacylglycerol
DNA: Deoxyribonucleic acid
DREB: Dehydration-responsive element-binding protein
GLRs: Glutamate receptors
GORK: Guard cell outward-rectifying K^+^ channel
H_2_O_2_: Hydrogen peroxide
HAK: High-affinity K^+^ uptake transporter
HKTs: High-affinity K^+^ transporters
IPs: Inositol phosphates
K^+^: Potassium
KOR: K^+^ outward-rectifying channels
MAPK: Mitogen-activated protein kinase
MDA: Malondialdehyde
MYB: Myeloblastosis oncogene
MYC: Myelocytomatosis oncogene
Na^+^: Sodium
NHX: Sodium-hydrogen exchanger proteins
NSCC: Nonselective cation channels
NSCCs: Nonselective cation channels
O_2_: Singlet oxygen
O^2-^: Superoxide radical
OH^−^: Hydroxyl ions
PM: Plasma membrane
PP2Cs: Phosphatases 2C
RbohF: Respiratory burst oxidase homolog protein F
ROS: Reactive oxygen species
SIMK: Salt stress-inducible MAPK
SIPK: Salicylic acid-induced protein kinase
SKOR: Stelar outward-rectifying K^+^ channel
SLAC1: Slow anion channel-associated1
SnRK2: Sucrose nonfermenting 1/SNF1-related protein kinase 2
SOS: Salt overly sensitive
TFs: Transcription factors.
